# Hepatic Sarcoid-Like Reaction Mimicking Liver Metastases in a 36-Year-Old Female With Rheumatoid Arthritis

**DOI:** 10.7759/cureus.43974

**Published:** 2023-08-23

**Authors:** Abdalla Khalil, Ahmed Taha

**Affiliations:** 1 Acute Medicine, Northwick Park Hospital, London North West University Healthcare NHS Trust, London, GBR; 2 Acute Medicine, Princess Royal University Hospital, King's College NHS Foundation Trust, London, GBR; 3 Medical Imaging, Princess Royal University Hospital, King's College Hospital NHS Foundation Trust, London, GBR; 4 Radiology, University of Manitoba, Winnipeg, CAN

**Keywords:** liver metastatis, liver metastases, hypercalcemia, anicteric cholestasis, etanercept, drug-induced sarcoidosis, hepatic secondaries

## Abstract

Hepatic and splenic sarcoidosis are still challenging issues for medical imaging, and in many cases, medical images can’t exclude the most common mimic of sarcoidosis which is liver metastases; therefore, a liver biopsy is required. A young female patient who had rheumatoid arthritis presented to our hospital with abdominal pain, anorexia, and weight loss for the past three weeks.

She was admitted to the acute medical ward and treated with intravenous fluid hydration for hypercalcemia. Her liver function tests were deranged (anicteric cholestasis picture), and her etanercept medication was stopped after being reviewed by the rheumatologist and gastroenterologist. She had a CT and MRI scan of the abdomen, an ultrasound (US) of the abdomen with enhanced contrast, and a positron emission tomography (PET) scan. The radiological findings could not exclude liver metastases, but an ultrasound-guided liver biopsy confirmed the finding of hepatic granulomatous changes of sarcoidosis. Her symptoms and hypercalcemia resolved, and her liver functions gradually normalized.

## Introduction

Sarcoidosis is a multisystem inflammatory disease of uncertain origin that is characterized by the formation of non-caseating granulomas. The most common respiratory manifestation is bilateral hilar adenopathy with or without changes of interstitial lung disease, and the common abdominal organs involved are the liver, the spleen, and the lymph nodes. The imaging findings of hepatic sarcoidosis usually consist of either homogeneous organomegaly or nodular infiltration, which are occasionally seen as lesions that can mimic liver metastases in both radiologic and scintigraphic studies [1]. Drug-induced sarcoidosis-like reactions (DISR) have the same clinical radiological findings as hepatic sarcoidosis but usually improve upon cessation of the incriminating drug.

## Case presentation

A 36-year-old female patient known to have rheumatoid arthritis presented to the hospital with lethargy, nausea, vomiting, abdominal pain, and weight loss for three weeks. Her rheumatoid arthritis symptoms were under control.

She looked emaciated and dehydrated and had mild right upper abdominal tenderness and mild distension. The rest of her clinical examination and observations (heart rate, temperature, respiratory rate, blood pressure, and pulse oxygen saturation) were within normal.

Her corrected serum calcium was 3.87 mmol/L (2.15-2.60 mmol/L), and phosphate was 0.66 mmol/L (0.80-1.40 mmol/L). Her liver function tests were deranged, revealing an "anicteric cholestasis picture".

Gamma glutamyl-transferase (GGT) was 420 U/L (5-40 U/L), alkaline phosphatase (ALP) was 123 IU/L (30-130 IU/L), alanine transaminase (ALT) was 76 IU/L (5-40 IU/L), and total bilirubin was 5 umol/L (5-20 umol/L). The parathyroid hormone was 8 ng/L (15-65 ng/L), and the C-reactive protein was 13 mg/L (0-5 mg/L).

The patient’s full blood count, renal profile, and thyroid function test were within the normal range. Her serum QuantiFERON-TB Gold (QFT) and COVID-19 polymerase chain reaction (PCR) tests were negative.

Her list of medications included sulfasalazine, etanercept (for nearly two years), and calcium supplements. She was reviewed by both the rheumatology and respiratory teams. She was admitted to the hospital and received an IV sodium chloride infusion (0.9%) and pamidronate. Both calcium supplements and etanercept were stopped.

**Figure 1:**
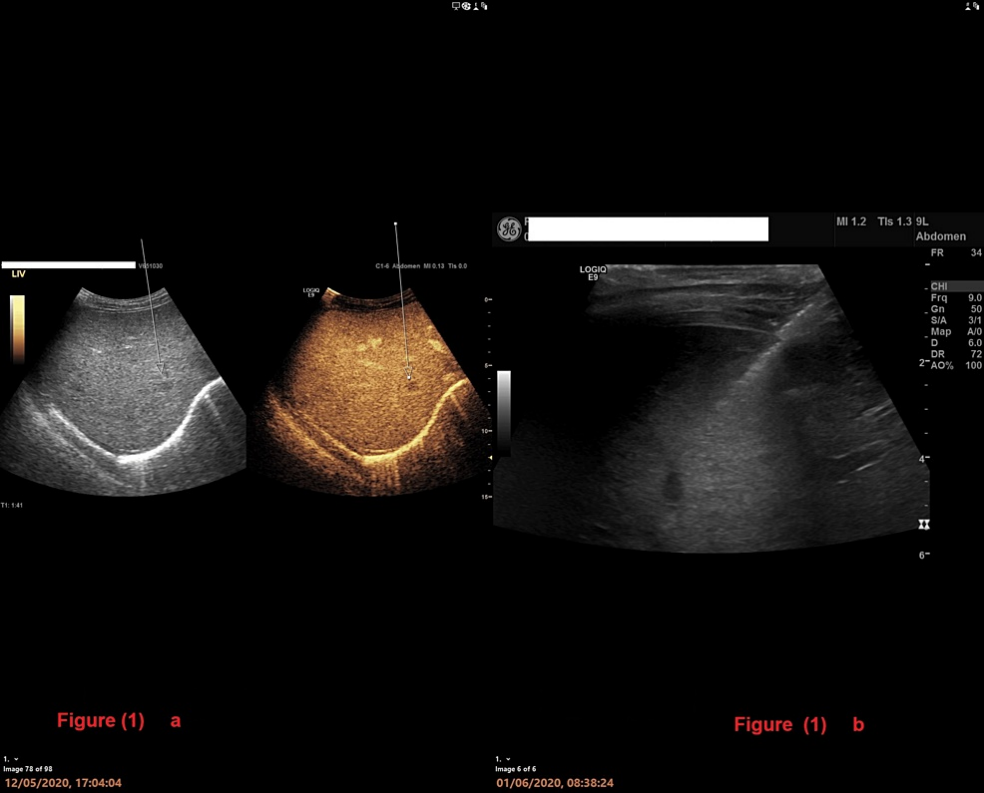
1a: A liver ultrasound (US) with contrast; 1b: US-guided liver biopsy 1a: US liver with contrast showed multiple focal lesions with hypo-enhancement; 1b: US-guided liver biopsy, which was performed after all the other medical imaging revealed histologically non-caseating granuloma and lymphocytic infiltration.

Her liver ultrasound with contrast showed multiple focal lesions with hypoenhancement (Figure 1a).

**Figure 2:**
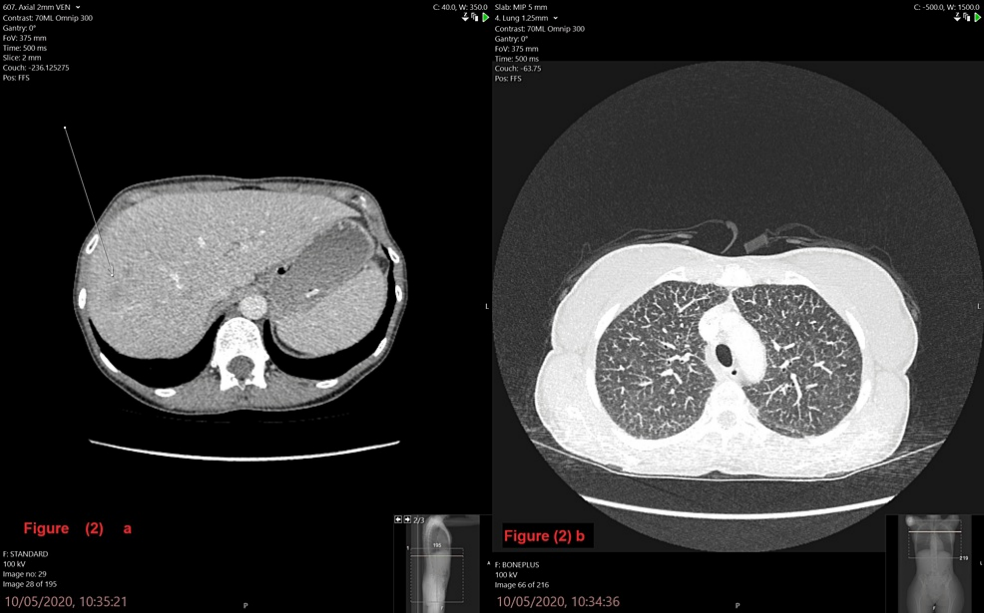
2a: CT scan of the patient's abdomen (axial view); 2b: CT scan of the chest (axial view) 2a: A CT of the abdomen (axial view) revealed multiple ill-defined hepatic lesions suggestive of metastasis vs. sarcoidosis, and the rest of the abdomen was normal; 2b: A CT scan of the chest (axial view) showed ground glass nodularity of the upper lobes and minor fissure nodules with no lymph node enlargement. These findings are most probably caused by sarcoidosis.

A CT scan of the patient’s abdomen (axial view) revealed multiple hepatic, ill-defined lesions suggestive of metastasis, and the rest of the abdomen was normal. A CT scan of the chest (axial view) showed ground-glass nodularity of the upper lobes and minor fissure nodules with no lymph node enlargement. These findings are most probably caused by sarcoidosis (Figure 2).

**Figure 3:**
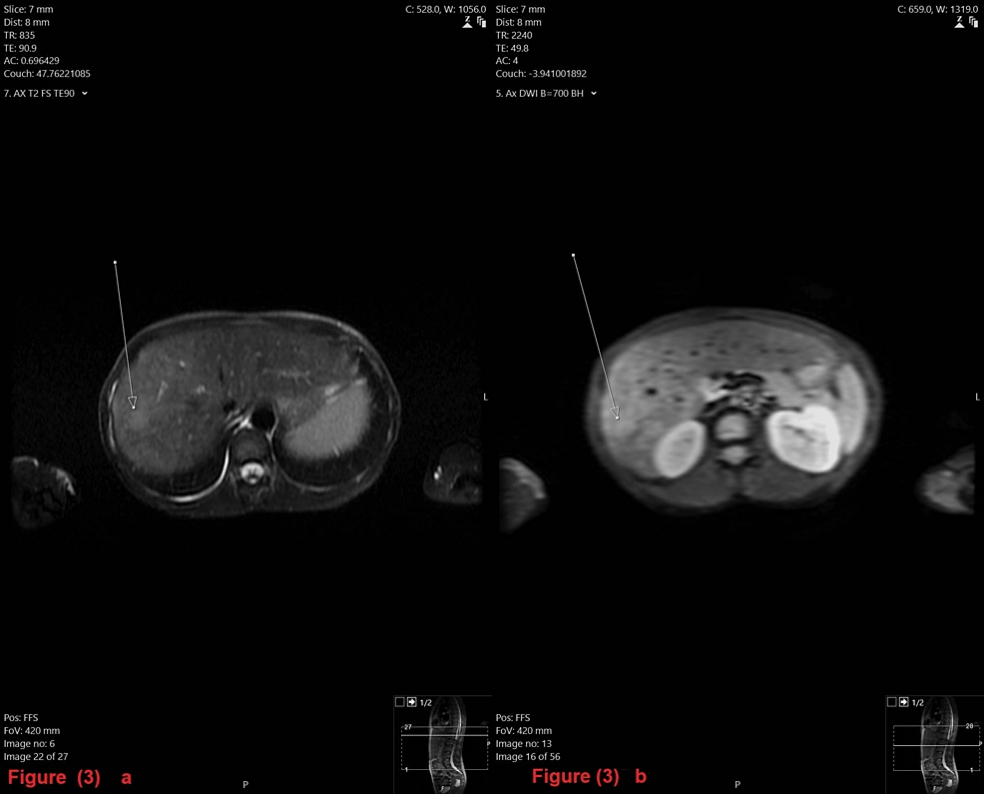
3a: MRI of the patient's abdomen (axial view) T2 weighted image (T2W1); Figure 3b: MRI of the abdomen (axial view) 3a: An MRI of the abdomen (axial view) T2WI revealed multiple lesions in the liver with hyperintensity, which appeared confluent in segments 7/8; 3b: An MRI of the abdomen (axial view) revealed multiple areas of restricted diffusion corresponding to the same lesions in the liver.

The MRI of her abdomen (axial view) T2 weighted image (T2W1) revealed multiple lesions in the liver with hyperintensity, which appeared confluent in segments 7/8. There were also multiple areas of restricted diffusion corresponding to the same lesions (Figures 3a-3b).

**Figure 4:**
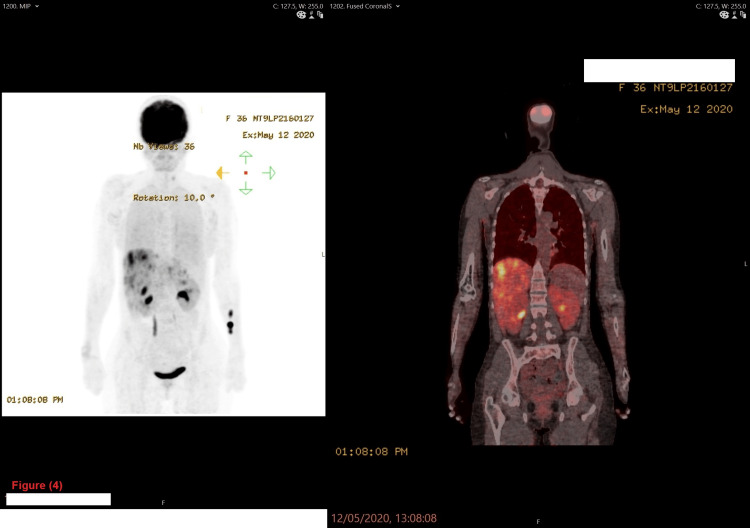
The patient's positron emission tomography (PET) scan (coronal view) The PET scan (coronal view) demonstrated an avid uptake of 2-deoxy-2-(18F)-fluorodeoxyglucose (FGD) by multiple lesions in the liver and one lesion in the spleen.

The positron emission tomography (PET) scan (coronal view) demonstrated an avid uptake of 2-deoxy-2-(18F)-fluorodeoxyglucose (FGD) by multiple lesions in the liver and one lesion in the spleen (Figure 4).

The multidisciplinary team decided to get a tissue sample to differentiate between the two possible diagnoses. The ultrasound-guided liver biopsy revealed histologically non-caseating granulomas and lymphocytic infiltration (Figure 1b).

Gradually, the patient improved clinically. Her serum calcium normalized, and her GGT and ALP came down, and she was discharged home.

## Discussion

Sarcoidosis is a rare granulomatous systemic disease that causes non-caseating granulomata. It most frequently involves the lungs and mediastinum, but hepatic involvement occurs in 50%-60% of patients [2]. Hepatic granulomas are diagnosed in 4% of total liver biopsies, and the common causes are systemic sarcoidosis, mycobacteria infections, Brucellosis, and primary biliary cholangitis [3]. Most patients with hepatic sarcoidosis are asymptomatic and investigated because of incidental deranged liver function tests. These liver function derangements are usually in the form of elevated ALP and GGT with normal bilirubin, i.e., "anicteric cholestasis". This pattern of liver function derangement is also common with liver metastases and medications [3, 4].

A drug-induced sarcoidosis-like reaction (DISR) is a systemic granulomatous reaction that is similar to sarcoidosis and usually improves after the withdrawal of the incriminating drug. A drug-induced sarcoidosis-like reaction can involve pulmonary and extra-pulmonary organs.

The list of DISR includes checkpoint inhibitors of cancer therapy (ipilimumab, nivolumab, and pembrolizumab), antiretroviral therapy, interferons (alpha and beta), and tumor necrosis factor-alpha (TNF-α) antagonists (like etanercept, adalimumab, and infliximab), as in our case presentation [5].

Radiologically, hepatic sarcoidosis may be difficult to distinguish from liver metastases and can only be differentiated by a liver biopsy [6-9]. Contrast-enhanced ultrasound (CEUS) and endoscopic ultrasound elastography are also used tools for detecting abdominal sarcoidosis, mainly hepato-splenic. Definite conclusions and recommendations are not possible so far because of the small number of patients investigated [10].

The most common initial presentations of pulmonary sarcoidosis are bilateral hilar lymphadenopathy or reticulonodular opacities. The initial presentation of sarcoidosis as pulmonary nodules is not uncommon [11]. Pulmonary nodules in sarcoidosis are poorly defined small nodules that are distributed along lymphatics, predictably localized along the pleural surfaces, fissures, and interlobular septae, and mainly involve the mid and upper lung fields [12].

## Conclusions

Sarcoid-like reactions can be induced by prolonged therapy groups of medication, including etanercept, which represents a medical image challenge because similar liver function derangement and medical image findings mimic liver metastases and require a liver biopsy for differentiation. The sarcoid-like reaction usually improves after the stoppage of the incriminating medications.
